# Diet and Foraging in the Waibira Chimpanzee Community, Budongo Central Forest Reserve, Uganda

**DOI:** 10.1002/ajp.70104

**Published:** 2025-12-13

**Authors:** Jakob Villioth, Jon Lim, Catherine Hobaiter, Klaus Zuberbühler, Nicholas E. Newton‐Fisher

**Affiliations:** ^1^ Living Primates Research Group University of Kent Canterbury UK; ^2^ Institute of Biology University of Neuchâtel Neuchâtel Switzerland; ^3^ Wild Minds Lab, School of Psychology and Neuroscience, University of St Andrews St Andrews Scotland; ^4^ Budongo Conservation Field Station Masindi Uganda

**Keywords:** Celtis, Ficus, Folivory, Frugivory, Pan troglodytes

## Abstract

Foraging is a fundamental aspect of the behavioural ecology of any species. Chimpanzees (*Pan troglodytes*) are generalist omnivores that inhabit a continuous range of forest environments. Accordingly, substantial differences in feeding ecology exist across chimpanzee sub‐species and populations. Despite a persistent importance for the role of ripe fruit, chimpanzee diets typically include a large variety of food types. While considerable data exist on the foraging behaviour and diets of chimpanzees, these are typically limited to studies of single communities in distinct populations. Previous studies in the Budongo forest, Uganda, have focused on the Sonso community; less is known of the foraging behaviour of the neighbouring Waibira community. Here, we present detailed descriptive data on diet, activity, and food availability from this community. These were collected between October 2016 and June 2017 from focal observations of ten adult males and nine adult females, phenological monitoring of 168 chimpanzee food trees, and 4 ha of botanical plots. These chimpanzees generally conformed to the view of this species as a ripe fruit specialist, but were notably less frugivorous than other study communities and showed a considerable reliance on young leaves, in particular the leaves of *Celtis mildbraedii*, and on the seeds of *Cynometra alexandrii* during the dry season. Dietary diversity was similar to that of the neighbouring Sonso community, and our results support the idea that significant folivory is a general foraging strategy for Budongo Forest chimpanzees.

## Summary

- Detailed descriptive data on the diet and food availability for a second, less studied community of chimpanzees in the Budongo Forest, Uganda
- Significant folivory is a general foraging strategy for Budongo Forest chimpanzees, suggesting a frugivore‐folivore designation

## Graphical abstract

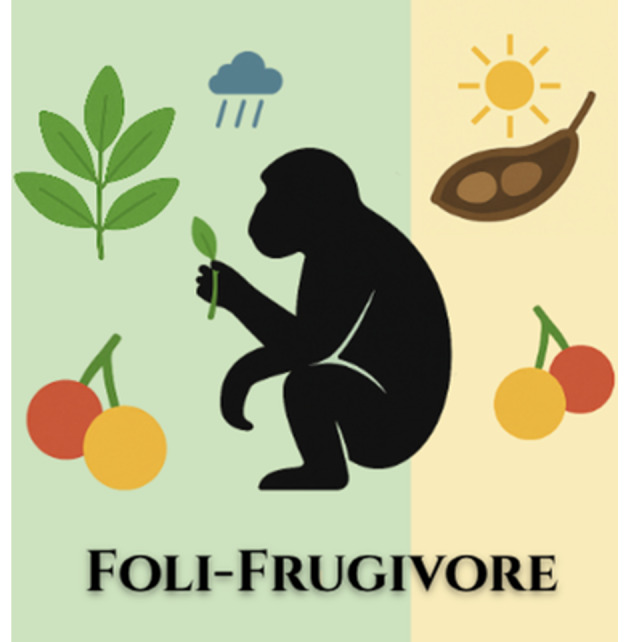

## 1 Introduction

Foraging is a fundamental aspect of the behavioural ecology of any species. The availability of food resources constrains female reproductive rates directly, and the reproductive rates of males indirectly where the number of potential mates is restricted by the number of individuals that a territory can support (Williams et al. 2004; Lemoine et al. 2020). Variation in food supply as a consequence of rainfall patterns, phenological patterns within and between plant (and prey) species, the behaviour and life‐history parameters of animal prey species, and succession processes within habitats, favour flexible foraging strategies and broad and variable diets, such as demonstrated by many primate species (Lambert 2007).

Chimpanzees (*Pan troglodytes* Blumenbach) are generalist omnivores that inhabit a continuous range of forest environments across Africa, from evergreen lowland rainforest (e.g. Taï National Park, Côte d'Ivoire, Boesch and Boesch‐Achermann 2000) to grassland–woodland–forest mosaics (e.g. Gombe National Park, Tanzania: Goodall 1986) and semi‐arid savanna grassland (e.g. Fongoli, Senegal: Pruetz and Bertolani 2007). Accordingly, substantial differences in feeding ecology exist across different chimpanzee sub‐species and populations, but their diets are typically dominated by ripe fruit (Wrangham 1986) and chimpanzees are thus sometimes described as “ripe fruit specialists” (Ghiglieri 1984; Watts et al. 2012a; Wrangham et al. 1998).

Despite a persistent importance for the role of ripe fruit, chimpanzees can include a large variety of food types in their diet, with young leaves providing – for example – important protein content (Carlson et al. 2013). Taï Forest chimpanzee supplement their diet by nut‐cracking during the dry season (Boesch and Boesch‐Achermann 2000); chimpanzees at Bossou in West Africa feed on oil‐palm kernels and oil‐palm pith in farmlands (Yamakoshi 1998); while in a semi‐arid, open environment in south‐eastern Senegal, the Fongoli community feeds on termites continuously throughout the year (Bogart and Pruetz 2008).

Studies of chimpanzees in the Budongo Forest, western Uganda, revealed termite feeding (Newton‐Fisher 1999a), the consumption of decaying wood (Reynolds et al. 2009; Reynolds et al. 2012; Reynolds et al. 2015), a high degree of folivory, and little reliance on terrestrial herbaceous vegetation (Newton‐Fisher 1997; Newton‐Fisher 1999b; Newton‐Fisher et al. 2000). While these findings drew contrasts with contemporaneous work on the Kanyawara community in the Kibale Forest, Uganda (Chapman et al. 1994; Wrangham et al. 1996) – which showed a greater degree of frugivory (80% of feeding time), little consumption of arboreal leaves (8% or less of feeding time), and a reliance on terrestrial herbaceous vegetation – more recent work (Bray et al. 2018) suggests a profile for Kanyawara very similar to that reported for Sonso (Kanyawara vs Sonso: ripe fruit: 65.3% vs 64.5%; arboreal leaves: 18.7% vs 19.7%), and a pattern (cf. Ngogo: Watts et al. 2012a; Kasekela & Mitumba: Gilby et al. 2017) of broadly similar dietary composition across East African chimpanzees (*P. t. schweinfurthii*).

Thus, considerable data exist on the foraging behaviour and diets of chimpanzees, but these have historically been limited to studies of single communities in distinct populations (Watts et al. 2012a), with comparisons between the Ngogo and Kanyawara communities being a notable exception (e.g. Potts et al. 2011). Previous studies of foraging and diet in the Budongo forest (Newton‐Fisher 1999b; Fawcett 2000; Tweheyo and Obua 2001) have focused on the Sonso community, which has been studied since the 1990s (Newton‐Fisher 1997; Reynolds 2005); less is known of the foraging behaviour of the neighbouring and more recently habituated Waibira community: this community shows distinctive differences in prey species preference (Hobaiter et al. 2017), and in behaviour around accessing water (Péter et al. 2022).

Although these communities are adjacent, their habitats are likely to differ floristically: the Budongo forest is a mosaic of vegetation types as a consequence of both commercial timber extraction from the 1920s to the 1960s (Babweteera et al. 2012; Plumptre 1996), and continuing illegal extraction of timber (Plumptre and Grieser Johns 2001) along with natural topographic variation. Much of the home range of the Waibira community was selectively logged more recently (1963–64 vs 1947–52), and more heavily, than were the areas ranged over by Sonso community chimpanzees (Plumptre 1996). We have shown previously that the two communities experience food patches of different sizes and distributions, with Waibira chimpanzees having access to a greater proportion of smaller – and fewer larger – food patches, typically travelling shorter distances between such patches (Villioth et al. 2022; Villioth et al. 2023). Here, we present more detailed descriptive data regarding diet, activity and food availability for the Waibira community, complementing our previously published studies. With a view to facilitating future comparative work, we draw comparisons with the adjacent Sonso and other, more distant, communities of East African chimpanzees.

## 2 Materials and Methods

### 2.1 Study Site and Community

Research was conducted within the Budongo Forest Reserve (1° 35' – 1° 55' N, 31° 08' – 31°42' E) in Western Uganda, 428 km² of medium‐altitude, moist, semi‐deciduous tropical forest. This forest is high in biodiversity, home to five species of diurnal primates including chimpanzees (*Pan troglodytes schweinfurthii*), and ranked third amongst protected areas in Uganda for species richness (Howard et al. 1997). The forest typically experiences a bimodal distribution of rain with a mean annual rainfall of around 1600 mm (Newton‐Fisher 1997; Reynolds 2005). Most rain falls between September and November, with a smaller rainy season between March and May. The dry season typically occurs between mid‐December and mid‐February (Newton‐Fisher 1999b). Temperatures vary significantly across months, with monthly maximums peaking at around 32°C during the dry season (Newton‐Fisher 1997).

At the time of this study, the Waibira community consisted of at least 88 known individual chimpanzees, including 17 adult males (≥ 16 years old) and 29 adult females (≥ 14 years old). Habituation of the Waibira chimpanzees started in 2011 (Samuni et al. 2014), and while this remains ongoing, almost all adult members could be individually recognized at the time of the study, followed for multiple consecutive hours, and remained neutral to observers at distances that permitted data on foraging behaviour to be collected (cf. Hobaiter et al. 2017). Data were collected from October 2016 to June 2017 by JV, together with the experienced field assistant Gideon Atayo.

### 2.2 Data Collection

We conducted focal follows from first light at the subject's night nest and continued for as long as conditions allowed. The focal animal was selected each day from a randomised list consisting of ten adult males and nine adult females. Males varied in age and represented different rank categories (high‐, mid‐, and low‐ranking). Seven of the females were lactating, while two females were not lactating but travelled with a single juvenile offspring. Ongoing habituation and the relatively dense habitat made continuous follows challenging (mean duration of follows: 4.1 ± 2.6 h; range: 1–12 h; median: 4 h; total: 491 h; follows < 1 h excluded from analysis). When an individual was lost, we attempted to increase the number of focal samples from individuals underrepresented in the overall sample in order to maintain a balanced sampling regime.

During focal follows, the behavioural state of the focal individual was recorded continuously (Altmann 1974) and categorized as either feeding (all behaviours related to food handling; the entire process of picking and ingesting food items), travelling (terrestrial quadrupedal walking as well as arboreal climbing and movement within the canopy), grooming (giving or receiving), resting (any period > 1 min in which the individual was sitting or lying and not engaging in another behaviour), or other (accounting for all other behaviours, e.g. copulations, play, drinking, etc.). GPS locations were recorded for all foraging sites, using a Garmin GPSMAP 64. For each food item consumed, we recorded the part (fruit, leaves, flowers, seeds, bark, soil, meat), species, and for plant foods, the phytophase (fruits: ripe, unripe; leaves: young, mature).

### 2.3 Ethical Note

This research complied with regulations set by the Ethics Committee of the University of Kent, the protocols of the Budongo Conservation Field Station (BCFS) and the legal requirements of Uganda, with permission granted by both the Ugandan Wildlife Authority (UWA) and the Ugandan National Council for Science and Technology (UNCST). This work complies with the American Society of Primatologists Principles for the Ethical Treatment of Non‐Human Primates, and the Code for Best Practices in Field Primatology. All fieldwork was observational, did not interfere with the chimpanzees, and followed disease transmission prevention protocols established by BCFS. The data that support the findings of this study are available from the corresponding author upon reasonable request.

### 2.4 Measures of Food Availability

Measuring food availability in tropical rain forests in a manner that is comparable across field sites and study species is notoriously difficult (Chapman et al. 1994). For this study, we chose to monitor fruit production of important arboreal food sources across 168 individual fruit trees, representing 14 important food species. Species were selected based on previous studies in Budongo (Newton‐Fisher 1999b; Fawcett 2000; Tweheyo and Obua 2001) and discussions with experienced Budongo Conservation Field Station field staff. The monitored trees, located across the home range of the study community, included 46 trees from four species of fig (*Ficus mucuso, F. sur, F. exasperata, F. variifolia*) as these have been identified previously as important food sources for chimpanzees in this population. For each monitored tree, we noted the presence of fruit (ripe and unripe), young leaves, flowers, and seeds on a monthly basis, following Chapman et al. (1994).

From these samples, we calculated three indices to represent monthly food availability: (1) FA_*fruit*_ : the conventional approach of considering only presence/absence of ripe fruit within each phenology‐monitored tree (Blake et al. 1990; Wrangham et al. 1998; for a review see Chapman et al. 1994); (2) FA_*leaves*_ : the young leaves of any species on which chimpanzees were observed to feed during each particular month, and (3) FA_*total*_ : ripe and unripe fruit, together with leaves, flowers, and seeds.

To assess the abundance of tree species and forest composition within the home range of the Waibira community, we established 10 botanical plots using a stratified random placement technique (Greig‐Smith 1983) across all habitat subtypes (see below) within the community's core area (Badihi et al. 2022). Habitat types were adapted from Plumptre (1996) and Newton‐Fisher (1997), and defined as follows:

1. *Primary forest*: Old growth mature forest, with little to no signs of human disturbance. Dominated by mature *Cynometra alexandrii*, and *Celtis mildbraedii*.
2. *Early‐ to mid‐stage regenerating forest*: selectively logged between 1963 and 1964, and still regenerating. Not dominated by a single species; mostly small, young trees. Canopy partly open.
3. *Wet forest* (valley bottom). Seasonally flooded.
4. *Swamp forest*. Permanently flooded. Swamp species, such as *Raphia farinifera*, present in the sample.

The home range of these chimpanzees is dominated by primary and regenerating forest, while wet and swamp forest are substantially localised (Plumptre 1996); thus four plots were located in each of the first two habitat types, and one in each of wet and swamp forest. Plots were constructed along pre‐existing trails and ran for 200 m along the trail. On 10 m to each side of the trail, all trees above 20 cm diameter at breast height (DBH) were identified and measured. Each plot was thus 200 x 20 m in size, resulting in a total surveyed area of 4 ha. We calculated the density (trees/ha) of all tree species that were identified within plots, and the basal area (m^2^/ha) for the top 15 food species to assess the availability and productivity of these important species (Chapman et al. 1994; Rode et al. 2006).

### 2.5 Data Analysis

Following convention, we classified food items as either high or low quality. Chimpanzees typically show a strong preference for ripe fruit, which offer a high content of easily digestible macronutrients such as non‐structural carbohydrates and lipids, and try to maintain a frugivorous diet even when fruit availability is low (Remis 2002; Wrangham et al. 1998). In the Budongo forest, seeds of *Cynometra alexandrii* also appear to be nutritionally valuable to chimpanzees as they are rich in lipids (Reynolds 2005). Thus, all ripe fruit, together with the seeds of *C. alexandrii*, were classified as high‐quality foods. Young leaves, flowers, and unripe fruit – which typically contain lower levels of sugars and higher levels of fibre and antifeedants (Houle et al. 2014; McLennan and Ganzhorn 2017) – were classified as low‐quality food.

To enable comparisons to investigations of other chimpanzee populations (Watts et al. 2012a; 2012b; Potts et al. 2011; Wrangham et al. 1998) and previous studies in Budongo (Newton‐Fisher 1999b; Fawcett 2000), we used the Shannon‐Wiener diversity index (Pielou 1975) to calculate a measure of dietary diversity:

$$H^{\prime }=-\sum (P_{i}{\text{.logP}}_{i})$$

where P_i_ is the proportion of species *i*. Exponentiating H′ generates a diversity measure, D (= Hill's N_1_), the ‘effective number of species’ (i.e. the number of equally‐common species that would produce the same value of H′), which is more comparable across studies (MacArthur 1965; Hill 1973; Jost 2006). We also calculated the standardized version of H′, to estimate dietary evenness:

$$J^{\prime }=H^{\prime }⁄\log(x)$$

where *x* is the total number of species sampled. Also known as Hill's (1973) equitability index, J′ indexes diversity on a 0–1 scale, with a score of 1 indicating that, in our application, the chimpanzees allocated their feeding time equally across dietary items.

### 2.6 Foraging Range

We recorded 430 GPS foraging locations, which we used to estimate foraging range during the study period for the Waibira chimpanzees. We employed two techniques: minimum convex polygons and kernel analysis. A minimum convex polygon is the smallest area polygon to encompass all of an animal's locations, producing an empirical estimate of range size but potentially biased by peripheral locations. Kernel analysis (Worton 1989; Seaman and Powell 1996) instead models the home range using the observed locations. We employed two methods, fixed and adaptive: fixed kernel analysis treats all data points equally, whereas adaptive kernel analysis varies the weight attached to each data point in relation to density (Gregory 2017). We used Ranges 9 (Kenward et al. 2014) to implement these methods.

### 2.7 Statistical Analysis

Statistical analyses were conducted in R versions 3.4.3 (R Core Team 2017). To calculate diversity indices, we used the *vegan* package (Oksanen et al. 2013). The Coefficient of Variation (CV) – the ratio of the standard deviation to the mean – we calculated from monthly values; associations between variables were investigated using Pearson correlations. We used a one‐tailed t‐test to address the hypothesis that male chimpanzees had larger foraging ranges than did females, drawing on prior findings from the neighbouring Sonso community (Bates and Byrne 2009).

## 3 Results

Chimpanzees of the Waibira community foraged during 35.9% of total observation time (491 h of focal animal sampling). Throughout the observation period (October 2016 – June 2017) they consumed parts of more than 31 different identified plant species, as well as young leaves and fruit from five or more unidentified species of liana. The mean number of (plant) food species consumed per month was 13 (range: 8–19). No single species accounted for more than 13% of feeding time, although collectively three species accounted for 35% of feeding time and ten accounted for 74% of feeding time (Table 1). Feeding on fruit accounted for 58.8% of feeding time (ripe fruit: 54.7%, unripe fruit: 4.1%), with *Ficus sur* being the most important species providing ripe fruit, while seeds of *Cynometra alexandrii* (the Uganda Ironwood) were also an important major food source during the dry season (Jan: 43%, Feb: 26%, Mar: 31%), and the second most consumed food item (11.83% of total feeding time). Leaves were consumed for 22.1% of feeding time (21.7% young leaves, 0.4% mature leaves), with young leaves of *Celtis mildbraedii* the most heavily consumed food item, accounting for 12.9% of the total feeding time, although these were not consumed at all during the dry season. One quarter of total feeding time was spent consuming either these leaves or the seeds of *Cynometra alexandrii*. The Waibira chimpanzees notably also consumed young leaves from two fig species (*F. exasperata, F. variifolia*), and unripe – as well as ripe – fruit of four fig species (*F. mucuso, F. sur, F. exasperata, F. variifolia*) in their top ten food species; at least one fig species was in the top 3 food species for seven of the 8 months of this study. Feeding on items designated as high quality (ripe fruit, seeds of *C. alexandrii*) never dropped below 44% of feeding time, and the proportion of time allocated to feeding remained largely unchanged month‐to‐month (CV = 0.10), despite changes in the species consumed.

**Table 1:** Plant species consumed by Waibira community chimpanzees, Budongo Forest, Uganda, and accounting for 0.5% or more of total feeding time.

Species	% of total feeding time	Plant part(s) consumed
*Celtis mildbraedii*	12.89	Young Leaves
*Cynometra alexandrii*	11.83	Seeds
*Ficus sur*	10.17	Ripe & Unripe Fruit
*Ficus mucuso*	8.00	Ripe & Unripe Fruit
*Drypetes gerrardii*	7.03	Ripe Fruit
*Chrysophyllum albidum*	5.76	Ripe Fruit
*Ficus saussureana*	4.90	Ripe Fruit
*Ficus exasperata*	4.71	Ripe & Unripe Fruit, Young Leaves
*Antiaris toxicaria*	4.44	Ripe Fruit
*Ficus variifolia*	4.16	Ripe & Unripe Fruit, Young Leaves
*Ficus polita*	2.76	Ripe Fruit
*Celtis durandii*	2.48	Ripe Fruit
*Maesopsis eminii*	2.23	Ripe Fruit
*Morus lactea*	1.73	Ripe & Unripe Fruit, Flowers
*Myrianthus holstii*	1.36	Ripe Fruit
*Ficus natalensis*	0.99	Ripe Fruit
*Cordia millenii*	0.93	Ripe & Unripe Fruit
*Chrysophyllum muerense*	0.85	Ripe Fruit
*Lasiodiscus mildbraedii*	0.78	Young Leaves, Flowers
*Desplatsia dewevrei*	0.73	Ripe & Unripe Fruit
*Celtis wightii*	0.62	Young Leaves
*Alaphia sp*.	0.51	Ripe Fruit

We recorded no instances of monkey predation, but did observe consumption of meat from two species of duiker (*Philantomba (Cephalophus) monticola*, *Cephalophus natalensis*), totalling 0.28% of feeding time. Feeding on terrestrial herbaceous vegetation (THV) amounted to only 0.67% of observed feeding time. The home range of the Waibira community does not border any cultivated fields, and these chimpanzees did not engage in any crop‐foraging.

Food availability as determined from our 168 monitored trees varied across months (Table 2). This was notable for both ripe fruit (FA_*fruit*_ CV = 0.72), and young leaves (FA_*leaves*_ CV = 1.08), but was more stable when considered more broadly (FA_*total*_ CV = 0.45). These species constituted 65% of the observed diet by feeding time, and 75.8% of feeding time for figs. Availability of young leaves declined markedly during the dry season, as might be expected given the semi‐deciduous nature of this habitat. Availability of ripe fruit peaked in April 2017 with species such as *Chrysophyllum albidum*, *Ficus sur*, *Myrianthus holstii*, and *Maesopsis eminii*, but excluding this month minimally impacts the estimation of the availability of ripe fruit (FA_*fruit*_ CV = 0.65). Ripe fruit was in some months driven by a single large tree: in December 2016, for example, a single *Ficus saussureana* was responsible for 35% of feeding time, with chimpanzees returning repeatedly over the period of one week.

**Table 2:** Dietary diversity and food availability for the Waibira community of chimpanzees in the Budongo forest, Uganda, October 2016 – May 2017. Food availability is expressed as the percentage of trees containing ripe fruit (FA_*fruit*_), percentage containing young leaves (FA_*leaves*_), and all potential exploited resources (FA_*total*_).

	Hʹ	Jʹ	D	FA_*fruit*_	FA_*leaves*_	FA_*total*_
October	1.61	0.63	5.00	1.19	3.57	10.71
November	2.11	0.82	8.25	2.98	7.14	12.50
December	2.1	0.76	8.17	1.19	4.17	11.31
January	1.93	0.78	6.89	6.55	0.00	17.26
February	2.19	0.83	8.94	10.12	0.00	13.86
March	1.81	0.87	6.11	7.74	0.00	15.66
April	2.5	0.85	12.18	13.11	13.86	32.53
May	1.34	0.54	3.82	4.17	7.27	26.67
Mean ± SD	1.95 ± 0.36	0.76 ± 0.12	7.42 ± 2.59	5.88 ± 4.3	4.50 ± 4.8	17.56 ± 7.9
Median	2.02	0.8	7.53	5.36	3.87	14.76

Mean dietary diversity (D) was 7.42 ± 2.59 effective species if calculated monthly (Table 2), or 7.03 effective species if determined from the monthly mean value of H′: this second value is more easily compared across published studies that provide summary values of H′ in preference to per‐month values (Table 3). Dietary diversity did not vary with overall food availability (FA_*total*_ : *r* = 0.30, *n* = 8, *p* = 0.47), although our data did suggest that dietary diversity might increase with the availability of ripe fruit (FA_*fruit*_ : *r* = 0.67, *n* = 8, *p* = 0.07). Feeding time was not related to dietary diversity for either ripe fruit (*r* = 0.28, *n* = 8, *p* = 0.50) or young leaves (*r* = –0.368, *n* = 8, *p* = 0.37).

**Table 3:** Inter‐community comparison of chimpanzee dietary diversity across four communities from two Ugandan forests, Budongo and Kibale.

Community	Hʹ	Jʹ	D	# Species/month	Source
Waibira	1.95 ± 0.13	0.76 ± 0.04	7.0	13 (8‐19)	*this study*
Sonso	1.80 ± 0.31	0.69 ± 0.09	6.1	13 (6‐19)	Newton‐Fisher (1999b)
Sonso	1.26 ± 0.38	0.61 ± 0.15	3.8	8 (6‐13)	Fawcett (2000)
Sonso	1.61 ± 0.03	0.67 ± 0.03	5.0	12 (6‐21)	Villioth (2018)
Ngogo	2.10 ± 0.39	0.65 ± 0.10	8.2	*nd*	Watts et al. (2012a)
Ngogo	1.55 ± *nd*	0.58 ± *nd*	4.7	*nd*	Potts et al. (2011)
Kanyawara	1.78 ± *nd*	0.70 ± *nd*	5.9	*nd*	Potts et al. (2011)

Dietary diversity was broadly similar to that of the neighbouring Sonso community (as assessed in both 1994–95 & 2015–16), although Waibira chimpanzees allocated 60.6% of feeding time to their seven most exploited species, compared to the Sonso chimpanzees' 64.9% on four species in 1994–95 (Newton‐Fisher 1999b) and 62.8% on five species in 2015–16 (Villioth 2018). The number of species consumed per month was comparable, although Waibira chimpanzees distributed their feeding time more evenly (Table 3).

Botanical plots (*n* = 10) contained 50–88 trees (mean ± SE = 75.9 ± 3.88) for a total of 750 trees of 70 different species. *Celtis mildbraedii* (*n* = 167, 22%) and *Cynometra alexandrii* (*n* = 93, 12.4%) were the most common trees within these plots (Table 4), and more common than in the adjacent Sonso region (*Celtis mildbraedii* 41.75 vs. 9.5 trees ha^‐1^; *Cynometra alexandrii* 23.25 vs 8.5 trees ha^‐1^: Villioth 2018). These were sizable, if not mature, trees (mean DBH = 40–50 cm). Thus, Waibira community chimpanzees foraged through largely *Celtis*‐dominated mixed‐forest. Of the 750 trees in these plots, 333 (44%) were of the top 15 food species for chimpanzees of this community (Table 5). Sixty‐two percent of trees in these plots were those on which chimpanzees fed for ≥ 0.5% of feeding time: 28% percent were of species that provided ripe fruit and seeds, while 34% provided young leaves and flowers, predominately *C. mildbraedii*. Twenty‐three percent of trees in these plots were non‐feeding trees. Figs were represented by 11 trees across four *Ficus* species, but neither *F. mucuso* or *F. saussureana* – important food species for Budongo forest chimpanzees – were present, reflecting their scarcity and localised distribution.

**Table 4:** Arboreal floristic composition of the Waibira community home range, as determined by botanical plots (*n* = 10, 200 m x 20 m ea.).

Species	*n*	%	Density (n/ha)	Average DBH (cm, mean ± SE)
*Alangium chinense*	8	1.1	2	30.78 ± 1.94
*Albizia glaberrimes*	8	1.1	2	73.5 ± 5.86
*Albizia zygia*	1	0.1	0.25	42.34
*Alstonia boonei*	7	0.9	1.75	63.93 ± 12.94
*Aningeria altissima*	5	0.7	1.25	45.4 ± 14.41
*Antiaris toxicaria*	9	1.2	2.25	29.71 ± 2.56
*Belonophora hypoglauca*	1	0.1	0.25	23.87
*Bosquea phoberos*	4	0.5	1	48.41 ± 8.07
*Caloncoba schweinfurthii*	7	0.9	1.75	26.01 ± 1.97
*Celtis africana*	2	0.3	0.5	30.24 ± 4.78
*Celtis durandii*	36	4.8	9	36.97 ± 2.2
*Celtis mildbraedii*	167	22.3	41.75	39.21 ± 1.4
*Celtis wightii*	2	0.3	0.5	21.65 ± 1.59
*Celtis zenkeri*	25	3.3	6.25	31.45 ± 2.15
*Chrysophyllum albidum*	6	0.8	1.5	47.23 ± 7.44
*Chrysophyllum muerense*	6	0.8	1.5	57.41 ± 2.99
*Chrysophyllum perpulchrum*	5	0.7	1.25	45.9 ± 11.48
*Cordia millenii*	5	0.7	1.25	44.37 ± 11.97
*Croton sylvaticus*	33	4.4	8.25	34.91 ± 2.02
*Cynometra alexandrii*	93	12.4	23.25	49.04 ± 2.27
*Desplatsia dewevrei*	1	0.1	0.25	19.74
*Diospyros abyssinica*	2	0.7	0.5	29.76 ± 2.39
*Dombeya mukole*	1	0.1	0.25	40.11
*Drypetes sp*.	1	0.1	0.25	24.83
*Ehretia cymosa*	3	0.4	0.75	27.27 ± 6.22
*Entandrophragama angolense*	1	0.1	0.25	20.37
*Erythrina excelsa*	1	0.1	0.25	21.65
*Erythrophleum suaveolens*	2	0.7	0.5	77.9 ± 10.49
*Fagara angolensis*	4	0.5	1	29.6 ± 3.98
*Fagaropsis angolensis*	2	0.7	0.5	40 ± 15
*Ficus exasperata*	5	0.7	1.25	53.9 ± 7.65
*Ficus polita*	1	0.1	0.25	40
*Ficus sur*	4	0.53	1	45.84 ± 15.37
*Ficus variifolia*	1	0.13	0.25	70
*Funtumia africana*	2	0.27	0.5	23.55 ± 3.82
*Funtumia elastica*	53	7.07	13.25	24.91 ± 0.58
*Greenwayodendron suaveolens*	1	0.13	0.25	28.58 ± 1.81
*Guarea cedrata*	1	0.13	0.25	32.79
*Harungana madagascariensis*	1	0.13	0.25	28.97
*Holoptelea grandis*	2	0.27	0.5	54.78 ± 10.22
*Khaya anthotheca*	5	0.67	1.25	47.3 ± 10.22
*Klainedoxa gabonensis*	1	0.13	0.25	33.42
*Lasiodiscus mildbraedii*	63	8.4	15.75	22.36 ± 0.45
*Leptaulus daphnoides*	5	0.67	1.25	28.2 ± 3.81
*Lychnodiscus cerospermus*	2	0.27	0.5	27.86 ± 4.62
*Macaranga monandra*	10	1.33	2.5	34.11 ± 4.1
*Macaranga schweinfurthii*	1	0.13	0.25	95
*Maerua duchesnei*	7	0.93	1.75	22.78 ± 1.52
*Maesopsis eminii*	5	0.67	1.25	57.55 ± 6.41
*Margaritaria discoideus*	19	2.53	4.75	45.32 ± 3.5
*Markhamia platycalyx*	3	0.4	0.75	36.29 ± 4.2
*Mildbraediodendron excelsum*	1	0.13	0.25	20.69
*Milettia spp*.	3	0.4	0.75	45.31 ± 13.61
*Monodora angolensis*	2	0.27	0.5	31.04 ± 7.17
*Morus lactea*	1	0.13	0.25	79.58
*Myrianthus holstii*	5	0.67	1.25	23.36 ± 1.33
*Paropsia guineensis*	5	0.67	1.25	45.71 ± 4.76
*Ricinodendron heudelotii*	3	0.4	0.75	51.46 ± 18.97
*Rinorea ardisiaeflora*	3	0.4	0.75	22.18 ± 1.54
*Sterculia dawei*	1	0.13	0.25	65
*Strombosia sp*.	1	0.13	0.25	23.87
*Strychnos mitis*	33	4.4	8.25	45.93 ± 3.77
*Tapura fischeri*	10	1.33	2.5	21.74 ± 0.85
*Teclea nobilis*	4	0.53	1	22.6 ± 1.59
*Tetrapleura tetraptera*	4	0.53	1	32.31 ± 5.18
*Tetrorchidium didymostemon*	5	0.67	1.25	39.28 ± 6.4
*Trema orientalis*	2	0.27	0.5	35.02 ± 3.19
*Trichilia dregeana*	2	0.27	0.5	27.53 ± 5.57
*Trichilia prieuriana*	8	1.07	2	22.1 ± 1.13
*Uvariopsis congensis*	6	0.8	1.5	22.07 ± 0.91
unknown	11	1.47	2.75	30.06 ± 5.48

**Table 5:** Abundance of the top 15 food species for Waibira chimpanzees as recorded within botanical plots (*n* = 10, 200 m x 20 m ea.).

Species	*n*	%	Density (n/ha)	Basal area (m^2^/ha)	Average DBH (cm, mean ± SE)	Part(s) Consumed
*Celtis mildbraedii*	167	22.3	41.75	6.11	39.21 ± 1.4	Young leaves, flowers
*Cynometra alexandrii*	93	12.4	23.25	5.26	49.04 ± 2.27	Seeds
*Ficus sur*	4	0.5	1	0.22	45.84 ± 15.37	Ripe/unripe fruit
*Ficus mucuso*						Ripe/unripe fruit
*Drypetes gerrardii*						Ripe fruit
*Chrysophyllum albidum*	6	0.8	1.5	0.3	47.23 ± 7.44	Ripe fruit
*Ficus saussureana*						Ripe fruit
*Ficus exasperata*	5	0.7	1.25	0.31	53.9 ± 7.65	Ripe/unripe fruit, young leaves
*Antiaris toxicaria*	9	1.2	2.25	0.17	29.71 ± 2.56	Ripe fruit, flowers
*Ficus variifolia*	1	0.1	0.25	0.1	70	Ripe/unripe fruit, young leaves
*Ficus polita*	1	0.1	0.25	0.03	40	Ripe fruit
*Celtis durandii*	36	4.8	9	1.09	36.97 ± 2.2	Ripe fruit
*Maesopsis eminii*	5	0.7	1.25	0.34	57.55 ± 6.41	Ripe fruit
*Morus lactea*	1	0.1	0.25	0.12	79.58	Ripe/unripe fruit, flowers
*Myrianthus holstii*	5	0.7	1.25	0.05	23.36 ± 1.33	Ripe fruit, young leaves

Foraging ranges covered an area of approximately 9 km^2^ (minimum convex polygon = 8.79 km^2^; fixed kernels = 8.94 km^2^; adaptive kernels = 10.00 km^2^). The core foraging range (50% fixed kernel estimate) during this period was 2.46 km^2^. Foraging ranges (fixed kernel estimates) peaked in size in the late dry season, although their size was not correlated with changes in food availability (vs. FA_*total*_ : *r* = −0.20, *n* = 8, *p* = 0.63; vs. FA_*fruit*_ : *r* = 0.35, *n* = 8, *p* = 0.39). Our sample suggests a possible negative relationship with the availability of young leaves (*r* = −0.64, *n* = 8, *p* = 0.09), which would match with the dietary shift from young leaves to *Cynometra alexandrii* seeds during the dry season.

Across communities of East African chimpanzees, adult male chimpanzees typically range more widely than do non‐cycling adult females (Tutin 1979; Goodall 1986; Nishida and Hiraiwa‐Hasegawa 1987), while Bates and Byrne (2009) suggest that Budongo Forest chimpanzee males explicitly combine foraging and territorial defence. Thus, and as predicted, Waibira community males had larger foraging ranges than did females (*t* = 1.787, df = 17, *p* < 0.05, one‐tailed; mean difference = 1.53; 95% CI: −0.27–3.34; *d* = 0.69). However, activity budgets of males and females were very similar: the difference between males and females did not exceed 3% in any of the behavioural categories (males vs. females: feeding: 36% vs. 37%; resting: 28% vs. 31%; grooming: 15% vs. 12%; travelling: 21% vs. 20%).

## 4 Discussion

As with chimpanzees elsewhere, the Waibira community generally conforms to the view of this species as a ripe fruit specialist (Ghiglieri 1984; Watts et al. 2012a; Wrangham et al. 1998): they spent the majority of their feeding time consuming ripe fruit, and maintained a relatively high proportion of feeding time on ripe fruit despite strong variation in its abundance. However, their percentage of time allocated to ripe fruit consumption was somewhat lower than reported for other *P. t. schweinfurthii* communities: 54.7% vs 65%–70% for Sonso (Newton‐Fisher 1999b; Fawcett 2000), 70.7% for Ngogo (Watts et al. 2012a; cf. 87% reported by Potts et al. 2011), 65%–80% for Kanyawara (Chapman et al. 1994; Wrangham et al. 1996; Bray et al. 2018), 64% for Kasekela, and 61% for Mitumba (Gilby et al. 2017). This figure (54.7%) is not much greater than the minimum cutoff (50%) for the definition of a dietary specialist (Pineda‐Munoz and Alroy 2014).

In addition, the Waibira chimpanzees showed a considerable reliance on young leaves, regularly consuming young leaves of several different tree species (22.1% of feeding time, cf. 19.7% for Sonso, Newton‐Fisher 1999a; 19.6%, Ngogo, Watts et al. 2012a; 18.7% for Kanyawara, Bray et al. 2018). Young leaves of *C. mildbraedii* were the single most consumed food item (at least in terms of feeding time) for Waibira chimpanzees across the study, as well as being consumed in large quantities when ripe fruit was scarce: these chimpanzees foraged exclusively on the young leaves of *C. mildbraedii* for several consecutive days when fruit was unavailable.

Young leaves are particularly rich in protein (Hladik 1977; Evans et al. 2021), and chimpanzees show both physiological (Dominy and Lucas 2001) and behavioural (Carlson et al. 2013) adaptations that appear specific to maximising the dietary benefits of young leaves. Further, Uwimbabazi et al. (2021) found evidence that chimpanzees prioritized available protein in their dietary choices. The leaves of *C. mildbraedii* – which contain no biologically active tannins (Evans et al. 2021) – are also recorded as being a highly preferred item for chimpanzees of the neighbouring Sonso community (Fawcett 2000), for whom young leaves (and flowers) of *Broussonetia papyrifera* also account for a substantial part of their diet (Newton‐Fisher 1999b; Villioth 2018). We have shown previously (Villioth et al. 2023) that chimpanzees of both Waibira and Sonso communities frequently chose to forage on food patches providing young leaves – patches of ripe fruit were not chosen in preference to those of young leaves – highlighting the importance of this food type in their diet.

Thus, our findings support the idea that significant folivory is an important aspect of the foraging strategy for Budongo Forest chimpanzees, demonstrated by both communities – despite local differences in ecology, with Waibira chimpanzees foraging in smaller and less dispersed patches (Villioth et al. 2022) – as is the reliance on the lipid‐rich seeds of *Cynometra alexandrii* during the dry season. Chimpanzee gut anatomy (Chivers and Hladik 1980) is characteristic of a frugivore‐folivore, and a rather coarse comparison across chimpanzee populations suggests that some are more frugivorous than others (citations above: e.g. up to 80% for Kanyawara vs. ~55% for Waibira). A research focus that assumes chimpanzees to be ripe‐fruit specialists may obscure a potential secondary specialisation on young leaves (as argued by Pineda‐Munoz and Alroy 2014), at least for some populations or at particular times. Ultimately, however, identifying how chimpanzees balance their nutritional demands (Uwimbabazi et al. 2021) across differing ecologies will be more significant than broad dietary categorisations.

Monthly food availability scores were comparatively low during our study, which may have been related to the atypically low rainfall: even during the usual wet season, the amount of rainfall (not greater than 200 mm) was more similar to typical months of ‘inter‐rains’ for this forest (Newton‐Fisher 1999b; Tweheyo and Lye 2003) and seasonality was not pronounced. Thus our findings represent, at best, a snap‐shot of the diet of these chimpanzees, and caution is warranted with regard to overgeneralising. Low availability of particular fruits upon which chimpanzees would otherwise feed may have reduced the degree of frugivory during the study period. For example, we expected greater consumption of *Strychnos mitis* fruit, but this was scarcely available to the chimpanzees during our study. However, such ‘atypical’ rainfall is a semi‐regular occurrence during, for example, *El Niño* events, and reflects conditions that these chimpanzees encounter repeatedly. Thus, it is important to understand feeding ecology during such periods. In addition, and despite a concerted effort to collect chimpanzee‐relevant phenological data, our choice of monitored trees did not include all those species selected by chimpanzees during the study. To the extent that chimpanzees’ diets track dynamic shifts in fruiting and leaf‐production – potentially at the level of individual trees – such a ‘phenology trail’ approach is always likely to lag, and so provide an incomplete solution to quantifying availability.

What is clear is that chimpanzees respond to the array of potential food items with which they are presented by adjusting their foraging behaviour, and that different food items, and species, are consumed differentially across seasons – or even days (Carlson et al. 2013) – individuals (depending on factors such as health, e.g. Freymann et al. 2024) and communities. Similarly, there is a need to consider diet wholistically – for instance, preference for particular food may depend on what else has been consumed that day (Freymann et al. 2024) – as well as in more detail and with regard to macronutrients (Rothman et al. 2012; Uwimbabazi et al. 2021) at the level of specific species, or even individual trees (Freymann et al. in review). If our goal is to understand the strategies – the decision rules – that underlie observed foraging behaviour (e.g. Villioth et al. 2023; Freymann et al. 2024), and the processes by which ecological variation drives grouping and social behaviour (e.g. Wrangham 1980; Wrangham 1986; Chapman et al. 1995; Sterck et al. 1997; Villioth et al. 2022), investigation into these dynamic responses should be central to future studies of diet and foraging behaviour, in both chimpanzees and other species.

## Author Contributions

**Jakob Villioth:** conceptualization (equal), formal analysis (lead), investigation (lead), methodology (equal), writing – original draft (lead). **Jon Lim:** formal analysis (supporting). **Catherine Hobaiter:** resources (equal), writing – review and editing (supporting). **Klaus Zuberbühler:** conceptualization (supporting), funding acquisition (equal), project administration (equal), resources (equal), supervision (equal), writing – review and editing (supporting). **Nicholas E Newton‐Fisher:** conceptualization (equal), formal analysis (supporting), funding acquisition (equal), methodology (equal), project administration (equal), resources (equal), supervision (equal), writing – original draft (supporting), writing – review and editing (lead).

## Data Availability

The data that support the findings of this study are available from the corresponding author upon reasonable request.
